# Supplementary material for: TSCytoPred: a deep learning framework for inferring cytokine expression trajectories from irregular longitudinal gene expression data to enhance multi-omics analyses
Source: PeerJ. 2025 Nov 10;13:e20270. doi: 10.7717/peerj.20270 (PMC12614104; doi:10.7717/peerj.20270)

Supplementary Material S3. Distribution of Spearman correlation coefficients across all cytokine-top 50 gene pairs in the COVID-19 dataset, with the dashed line indicating the average.

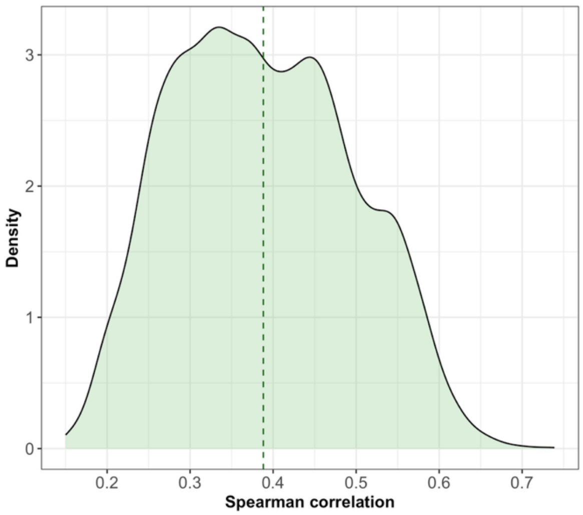

Supplement: Supplemental Information 3 [file peerj-13-20270-s003.pdf]
