# Supplementary material for: TSCytoPred: a deep learning framework for inferring cytokine expression trajectories from irregular longitudinal gene expression data to enhance multi-omics analyses
Source: PeerJ. 2025 Nov 10;13:e20270. doi: 10.7717/peerj.20270 (PMC12614104; doi:10.7717/peerj.20270)
Supplement: Supplemental Information 4 [file peerj-13-20270-s004.pdf]

Supplementary Material S4.

Sumarry statistics for Spearman correlation and corresponding p-values of the top 50 genes selected for each cytokine.

Cytokine	Spearman Correlation				p-value			
	Min	Max	Median	IQR	Min	Max	Median	IQR
IL12p70	0.5094	0.6362	0.5462	0.0353	4.69E-33	7.97E-20	4.30E-23	4.31E-21
TGFB2	0.3673	0.4345	0.3866	0.0281	2.82E-14	2.44E-10	2.24E-11	8.44E-11
CX3CL1	0.5161	0.6102	0.5395	0.0329	7.44E-30	2.15E-20	1.80E-22	1.06E-21
IL2	0.2438	0.3181	0.2631	0.0253	5.60E-08	3.85E-05	8.41E-06	1.95E-05
SERPINB4	0.2697	0.3378	0.2864	0.0194	7.10E-09	4.89E-06	1.14E-06	2.44E-06
NTF4	0.2475	0.3543	0.2625	0.0275	1.13E-09	2.90E-05	8.87E-06	1.68E-05
IL1A	0.2735	0.4412	0.2996	0.0329	1.02E-14	3.55E-06	3.42E-07	8.93E-07
MMP13	0.4623	0.5605	0.4991	0.042	1.77E-24	3.55E-16	5.60E-19	3.48E-17
MMP12	0.3445	0.5284	0.3998	0.0652	1.81E-21	3.42E-09	3.95E-12	3.37E-10
IL4	0.3051	0.3863	0.3178	0.0173	2.30E-11	2.02E-07	5.77E-08	1.08E-07
IFNG	0.3196	0.4111	0.3314	0.012	8.40E-13	4.83E-08	1.42E-08	1.70E-08
IGFBP4	0.2399	0.3159	0.258	0.0312	6.99E-08	5.15E-05	1.28E-05	2.81E-05
IL33	0.4055	0.5423	0.4374	0.0392	9.88E-23	1.81E-12	1.84E-14	5.35E-13
IL1B	0.5185	0.6478	0.5538	0.04	1.36E-34	1.33E-20	8.48E-24	8.93E-22
IL5	0.528	0.6385	0.5517	0.0312	2.37E-33	1.97E-21	1.31E-23	2.87E-22
TNFSF11	0.1793	0.3015	0.1955	0.0176	2.84E-07	0.0026536	0.00102605	0.00113103
THPO	0.3534	0.4913	0.3699	0.0279	2.34E-18	1.25E-09	1.80E-10	3.64E-10
CXCL1	0.3268	0.4116	0.3471	0.025	7.79E-13	2.30E-08	2.58E-09	7.22E-09
TSLP	0.3257	0.5026	0.36	0.0526	2.91E-19	2.58E-08	5.85E-10	5.79E-09
CXCL9	0.435	0.5769	0.4642	0.0387	3.73E-26	2.62E-14	2.56E-16	1.97E-15
LIF	0.3062	0.4515	0.3395	0.0461	2.03E-15	1.82E-07	5.92E-09	4.96E-08
CXCL11	0.544	0.6216	0.5624	0.0306	3.20E-31	6.91E-23	1.14E-24	8.04E-24
IL25	0.323	0.4719	0.361	0.0587	7.03E-17	3.39E-08	5.15E-10	3.52E-09
IL12B	0.415	0.5379	0.443	0.0366	2.52E-22	4.83E-13	7.66E-15	5.02E-14
IL10	0.4217	0.5099	0.4402	0.0243	7.21E-20	1.86E-13	1.19E-14	5.31E-14
IL13	0.4531	0.5442	0.4755	0.0337	6.50E-23	1.57E-15	3.80E-17	2.92E-16
IL11	0.2463	0.335	0.2581	0.0176	9.65E-09	3.19E-05	1.26E-05	1.70E-05
IL15	0.3563	0.5424	0.3986	0.0539	9.67E-23	8.99E-10	4.91E-12	1.37E-10
PTX3	0.5095	0.662	0.536	0.058	1.47E-36	7.75E-20	3.90E-22	1.38E-20
BMP10	0.5383	0.6355	0.5635	0.0278	5.83E-33	2.30E-22	9.36E-25	1.41E-23
TNFRSF13B	0.2221	0.2886	0.2393	0.0227	9.45E-07	0.00018405	5.41E-05	9.18E-05
VEGFA	0.3117	0.4037	0.3275	0.0255	2.31E-12	1.06E-07	2.14E-08	4.84E-08
IL3	0.3378	0.5113	0.3765	0.0678	5.53E-20	7.09E-09	8.03E-11	2.03E-09
CSF1	0.4551	0.5587	0.48	0.036	2.63E-24	1.13E-15	1.77E-17	2.00E-16
IFNL3	0.2267	0.3036	0.2425	0.0211	2.33E-07	0.0001338	4.26E-05	7.79E-05
IFNA	0.4567	0.5596	0.4909	0.0432	2.15E-24	8.81E-16	2.56E-18	1.52E-16
FGF2	0.2617	0.3818	0.2789	0.0225	4.12E-11	9.48E-06	2.23E-06	4.21E-06
GZMA	0.3084	0.4763	0.3406	0.0464	3.32E-17	1.46E-07	5.28E-09	4.23E-08
TGFA	0.4654	0.634	0.5036	0.0744	9.01E-33	2.09E-16	2.49E-19	9.31E-18
IL23A	0.4655	0.5584	0.4844	0.0426	2.85E-24	2.07E-16	8.08E-18	6.73E-17
GH1	0.1904	0.2488	0.2055	0.0225	2.63E-05	0.00139956	0.00055396	0.00077169
TNFSF10	0.2474	0.3407	0.2618	0.0372	5.22E-09	2.92E-05	9.38E-06	1.91E-05
CXCL13	0.3701	0.5207	0.3952	0.0518	8.68E-21	1.75E-10	7.26E-12	4.70E-11
TREM1	0.4412	0.6157	0.4701	0.0763	1.67E-30	1.02E-14	1.04E-16	1.51E-15
C1Q	0.2901	0.4187	0.3165	0.0361	2.85E-13	8.20E-07	6.63E-08	2.83E-07
CCL3	0.3595	0.5068	0.3862	0.0406	1.31E-19	6.21E-10	2.33E-11	1.61E-10
MICA	0.3594	0.4401	0.3733	0.0182	1.20E-14	6.24E-10	1.18E-10	2.57E-10
CXCL8	0.39	0.5423	0.422	0.0333	9.88E-23	1.44E-11	1.80E-13	1.12E-12
NECTIN4	0.332	0.4976	0.3761	0.0572	7.39E-19	1.32E-08	8.60E-11	1.17E-09
TNFSF13	0.4998	0.611	0.5269	0.0404	6.04E-30	4.85E-19	2.48E-21	8.95E-20
TNFSF14	0.4462	0.6084	0.4777	0.0378	1.22E-29	4.70E-15	2.62E-17	5.24E-16
KIT	0.2113	0.2664	0.2215	0.021	6.46E-06	0.00037925	0.00019132	0.00022265
IL6	0.4422	0.5443	0.4611	0.0279	6.48E-23	8.75E-15	4.27E-16	1.46E-15
TNF	0.5557	0.6871	0.5902	0.045	2.64E-40	5.22E-24	1.41E-27	1.97E-25

MIA	0.2389	0.352	0.2542	0.0291	1.47E-09	5.54E-05	1.73E-05	2.75E-05
TNFRSF10B	0.4755	0.6591	0.511	0.0774	3.83E-36	3.84E-17	7.38E-20	6.88E-18
KITLG	0.3349	0.4261	0.3502	0.0196	9.84E-14	9.71E-09	1.81E-09	4.75E-09
LEP	0.1991	0.294	0.2127	0.0345	5.72E-07	0.00082321	0.00034578	0.00057169
MMP7	0.1932	0.2866	0.2228	0.0406	1.13E-06	0.00118312	0.00017569	0.00065236
FCER2	0.15	0.2311	0.1632	0.0165	9.78E-05	0.01211583	0.00630457	0.00507864
NPHS1	0.3087	0.4703	0.3605	0.0837	9.27E-17	1.43E-07	5.98E-10	4.41E-08
ICAM1	0.2661	0.3427	0.2804	0.0182	4.15E-09	6.57E-06	1.98E-06	3.15E-06
MUC16	0.3781	0.4831	0.3943	0.039	1.01E-17	6.54E-11	8.18E-12	2.35E-11
IGFBP1	0.2572	0.349	0.2789	0.0286	2.07E-09	1.36E-05	2.23E-06	6.53E-06
CXCL5	0.3147	0.5215	0.3412	0.0445	7.38E-21	7.90E-08	4.92E-09	1.84E-08
B2M	0.4786	0.5799	0.4997	0.0415	1.83E-26	2.21E-17	5.03E-19	6.64E-18
A2M	0.2132	0.3448	0.2298	0.0229	3.30E-09	0.00033487	0.0001076	0.00017875
ANGPT2	0.3109	0.3855	0.3257	0.0214	2.55E-11	1.14E-07	2.58E-08	5.70E-08
ERBB2	0.1922	0.285	0.2013	0.0147	1.30E-06	0.00125825	0.00071967	0.00063147
CCL4	0.3996	0.5543	0.4298	0.0403	7.17E-24	4.02E-12	5.68E-14	1.02E-12
CEACAM1	0.3842	0.4706	0.3979	0.0219	8.87E-17	3.03E-11	5.10E-12	1.29E-11
CCL26	0.2768	0.3739	0.2956	0.0264	1.09E-10	2.66E-06	4.97E-07	1.56E-06
MBL2	0.2203	0.3121	0.2324	0.028	1.02E-07	0.00020794	8.93E-05	0.00012363
EGF	0.3375	0.4448	0.3564	0.0314	5.79E-15	7.35E-09	8.89E-10	3.56E-09
CHI3L1	0.3715	0.4597	0.3881	0.0242	5.43E-16	1.46E-10	1.84E-11	7.47E-11
CXCL10	0.5406	0.6525	0.5652	0.038	3.19E-35	1.42E-22	5.95E-25	1.79E-23
CXCL12	0.348	0.5313	0.3809	0.0424	1.00E-21	2.31E-09	4.60E-11	7.88E-10
OSM	0.3833	0.5711	0.4115	0.0568	1.48E-25	3.38E-11	8.08E-13	8.23E-12
TGFB1	0.3315	0.5233	0.3554	0.0434	5.16E-21	1.40E-08	9.96E-10	5.20E-09
FASLG	0.3397	0.4696	0.3652	0.0333	1.04E-16	5.79E-09	3.14E-10	9.81E-10
REG3A	0.3549	0.4283	0.3723	0.0259	7.15E-14	1.05E-09	1.33E-10	4.97E-10
VWF	0.3221	0.4192	0.3447	0.0265	2.69E-13	3.72E-08	3.33E-09	1.19E-08
PDGFA	0.3377	0.4868	0.3538	0.0236	5.28E-18	7.18E-09	1.20E-09	3.58E-09
ALDH1A1	0.3642	0.441	0.3859	0.0236	1.05E-14	3.54E-10	2.44E-11	1.15E-10
PLAUR	0.4767	0.5692	0.5065	0.035	2.37E-25	3.12E-17	1.38E-19	2.47E-18
PROC	0.2386	0.3065	0.2545	0.0271	1.76E-07	5.67E-05	1.69E-05	3.04E-05
MB	0.4243	0.546	0.453	0.0497	4.46E-23	1.29E-13	1.60E-15	8.53E-15
FLT1	0.3265	0.4564	0.3486	0.0312	9.17E-16	2.36E-08	2.17E-09	7.10E-09
MPO	0.4585	0.6142	0.4935	0.07	2.54E-30	6.54E-16	1.59E-18	5.91E-17
THBS2	0.3802	0.4619	0.3923	0.023	3.74E-16	5.02E-11	1.07E-11	2.64E-11
MUC1	0.3108	0.454	0.3358	0.0461	1.37E-15	1.15E-07	8.85E-09	3.83E-08
TIMP1	0.4833	0.6525	0.5135	0.0461	3.13E-35	9.84E-18	3.71E-20	5.39E-19
VCAM1	0.5371	0.6781	0.5711	0.0397	6.33E-39	2.97E-22	1.51E-25	1.34E-23
MMP2	0.1865	0.2592	0.2005	0.0186	1.16E-05	0.00175287	0.00075797	0.00089603
TNFRSF17	0.2648	0.3542	0.2801	0.0302	1.15E-09	7.35E-06	2.01E-06	3.50E-06
MMP9	0.4499	0.6044	0.4873	0.0502	3.58E-29	2.60E-15	4.76E-18	1.23E-16
MMP8	0.4383	0.5978	0.4749	0.0696	1.99E-28	1.58E-14	4.29E-17	1.37E-15
TNFSF13B	0.5333	0.6256	0.5478	0.0298	1.03E-31	6.60E-22	3.07E-23	1.88E-22
THBD	0.3235	0.4726	0.3534	0.0525	6.31E-17	3.24E-08	1.28E-09	7.60E-09
MMP3	0.3181	0.4591	0.3587	0.0447	5.95E-16	5.61E-08	6.75E-10	7.00E-09
PF4	0.2904	0.449	0.3125	0.0321	2.99E-15	7.97E-07	9.86E-08	3.17E-07
SFTPD	0.3751	0.5525	0.4165	0.0558	1.06E-23	9.51E-11	4.12E-13	1.32E-11
TFF3	0.2319	0.3035	0.2536	0.0285	2.34E-07	9.22E-05	1.81E-05	4.28E-05
SDC1	0.4184	0.5744	0.4656	0.0593	6.79E-26	3.00E-13	2.43E-16	1.59E-14
PECAM1	0.2477	0.3625	0.2672	0.0298	4.33E-10	2.87E-05	6.00E-06	1.54E-05
PDGFD	0.3506	0.5505	0.3793	0.0595	1.66E-23	1.72E-09	5.62E-11	5.83E-10
PRL	0.403	0.5023	0.4437	0.0358	3.07E-19	2.55E-12	6.91E-15	1.30E-13
MIF	0.4056	0.5077	0.4292	0.0313	1.10E-19	1.80E-12	6.20E-14	7.37E-13
RETN	0.3911	0.5551	0.4222	0.0562	5.98E-24	1.25E-11	1.74E-13	1.70E-12
S100A12	0.4315	0.5588	0.4656	0.0472	2.60E-24	4.44E-14	2.06E-16	3.87E-15
S100A9	0.4265	0.5565	0.461	0.0449	4.31E-24	9.29E-14	4.36E-16	1.09E-14
SELL	0.2401	0.2942	0.253	0.0233	5.64E-07	5.09E-05	1.89E-05	3.07E-05
ST2	0.5573	0.7389	0.5983	0.0701	2.10E-49	3.66E-24	1.94E-28	3.73E-26
PCSK9	0.3571	0.4342	0.3768	0.0257	2.93E-14	8.17E-10	7.72E-11	2.95E-10

SELP	0.321	0.4519	0.3371	0.0317	1.89E-15	4.18E-08	7.71E-09	2.23E-08
SERPINA10	0.4013	0.4634	0.414	0.0215	2.95E-16	3.20E-12	5.58E-13	1.05E-12
SERPINA4	0.4194	0.5454	0.4446	0.0263	5.06E-23	2.60E-13	6.00E-15	3.48E-14
SERPINC1	0.4172	0.5433	0.4441	0.032	7.95E-23	3.56E-13	6.49E-15	7.17E-14
PLAU	0.3	0.3905	0.3212	0.0198	1.35E-11	3.28E-07	4.07E-08	1.13E-07
SPP1	0.4521	0.5353	0.4693	0.0267	4.37E-22	1.84E-15	1.13E-16	5.38E-16
PROS1	0.2832	0.353	0.293	0.0234	1.30E-09	1.52E-06	6.37E-07	8.93E-07
IL1RN	0.4134	0.5384	0.4329	0.0283	2.26E-22	6.12E-13	3.59E-14	1.70E-13
MCAM	0.2325	0.3651	0.253	0.0191	3.20E-10	8.84E-05	1.90E-05	3.12E-05
CCL23	0.3079	0.3725	0.3206	0.029	1.30E-10	1.54E-07	4.32E-08	9.06E-08
CSF1R	0.2413	0.3357	0.258	0.02	8.95E-09	4.63E-05	1.28E-05	1.65E-05
COL1A1	0.2739	0.3885	0.3003	0.0429	1.75E-11	3.42E-06	3.20E-07	1.59E-06
CNTN1	0.2712	0.3808	0.2905	0.0219	4.68E-11	4.30E-06	7.90E-07	1.61E-06
CLEC11A	0.4644	0.5247	0.4809	0.0295	3.89E-21	2.50E-16	1.49E-17	4.78E-17
CFP	0.2632	0.3336	0.2749	0.0166	1.12E-08	8.39E-06	3.15E-06	4.51E-06
CFD	0.2576	0.3423	0.2774	0.0313	4.38E-09	1.32E-05	2.54E-06	6.45E-06
CD40LG	0.2877	0.4321	0.3068	0.0206	4.06E-14	1.02E-06	1.71E-07	4.53E-07
CD274	0.5315	0.6411	0.5561	0.0427	1.07E-33	9.61E-22	4.76E-24	2.07E-22
CD163	0.2497	0.3715	0.2705	0.0278	1.47E-10	2.46E-05	4.57E-06	1.10E-05
CD14	0.5184	0.5971	0.5427	0.0211	2.39E-28	1.35E-20	8.99E-23	1.09E-21
CCL5	0.2941	0.4485	0.3263	0.0718	3.27E-15	5.66E-07	2.42E-08	2.35E-07
CCL2	0.435	0.5444	0.4504	0.0283	6.34E-23	2.62E-14	2.43E-15	7.28E-15
CCL24	0.4864	0.6212	0.5322	0.0409	3.58E-31	5.67E-18	8.33E-22	9.70E-20
CCL22	0.3102	0.4073	0.3359	0.0274	1.42E-12	1.23E-07	8.95E-09	2.88E-08
CXCL6	0.2684	0.3586	0.2917	0.0255	6.89E-10	5.46E-06	7.13E-07	1.43E-06
CCL19	0.2837	0.3577	0.2991	0.03	7.58E-10	1.46E-06	3.60E-07	7.87E-07
CCL18	0.2411	0.3561	0.2621	0.0414	9.18E-10	4.71E-05	9.14E-06	2.53E-05
CCL14	0.2882	0.4389	0.3149	0.0375	1.45E-14	9.75E-07	7.72E-08	3.27E-07
CCL11	0.3098	0.4239	0.3294	0.0351	1.35E-13	1.28E-07	1.75E-08	5.20E-08
C9	0.4283	0.5141	0.4466	0.0367	3.20E-20	7.11E-14	4.43E-15	1.88E-14
C5	0.3102	0.3825	0.3296	0.0218	3.75E-11	1.22E-07	1.72E-08	5.53E-08
C2	0.4019	0.4811	0.4248	0.021	1.44E-17	2.97E-12	1.18E-13	6.21E-13
BDNF	0.2929	0.4191	0.3176	0.0516	2.71E-13	6.34E-07	5.91E-08	2.56E-07
AOC3	0.2253	0.3193	0.2552	0.0339	4.97E-08	0.00014705	1.60E-05	4.40E-05
ANGPTL3	0.2577	0.3365	0.2742	0.0199	8.20E-09	1.31E-05	3.35E-06	6.79E-06
ANGPT1	0.2908	0.4383	0.319	0.0381	1.59E-14	7.71E-07	5.12E-08	2.12E-07
AMBP	0.2197	0.3161	0.2414	0.0334	6.81E-08	0.00021759	4.67E-05	0.00014486
AHSG	0.3977	0.5045	0.4201	0.0247	2.01E-19	5.20E-12	2.35E-13	1.72E-12
CXCL2	0.3952	0.473	0.413	0.0282	5.87E-17	7.25E-12	6.46E-13	3.07E-12
DKK1	0.4097	0.5702	0.4504	0.0536	1.87E-25	1.02E-12	2.62E-15	7.27E-14
MADCAM1	0.2539	0.3378	0.2701	0.0224	7.13E-09	1.77E-05	4.71E-06	8.34E-06
IL18	0.3513	0.4642	0.3687	0.0308	2.59E-16	1.58E-09	2.08E-10	6.83E-10
LUM	0.1967	0.2734	0.213	0.0234	3.56E-06	0.00095468	0.00033983	0.00045385
LRG1	0.5029	0.5779	0.5235	0.0242	2.98E-26	2.71E-19	5.07E-21	6.67E-20
LGALS9	0.4153	0.5032	0.4346	0.0298	2.56E-19	4.63E-13	2.80E-14	1.83E-13
LGALS3	0.4564	0.6218	0.4938	0.0632	3.02E-31	9.18E-16	1.50E-18	1.20E-16
LGALS3BP	0.4741	0.5932	0.4943	0.0319	6.65E-28	4.83E-17	1.35E-18	1.03E-17
LCN2	0.4067	0.5471	0.4373	0.0437	3.48E-23	1.55E-12	1.85E-14	1.81E-13
LBP	0.5487	0.6585	0.5776	0.0337	4.66E-36	2.45E-23	3.49E-26	3.30E-24
KLK6	0.2735	0.3619	0.2918	0.0213	4.66E-10	3.53E-06	7.01E-07	1.51E-06
IL7	0.3675	0.5594	0.388	0.0365	2.27E-24	2.39E-10	1.86E-11	1.03E-10
IL6R	0.2862	0.3783	0.3018	0.0312	6.33E-11	1.17E-06	2.75E-07	5.26E-07
IL4R	0.4413	0.5362	0.458	0.0194	3.60E-22	1.00E-14	7.14E-16	3.17E-15
IL36B	0.3373	0.5356	0.3627	0.0583	4.08E-22	7.48E-09	4.26E-10	2.22E-09
ADAMTS13	0.2906	0.337	0.3031	0.0184	7.80E-09	7.84E-07	2.44E-07	4.94E-07
IGFBP2	0.3665	0.5217	0.398	0.0447	7.07E-21	2.70E-10	5.20E-12	8.72E-11
DPP4	0.576	0.6986	0.5897	0.0261	3.67E-42	4.62E-26	1.61E-27	6.95E-27
HGF	0.4745	0.6396	0.5086	0.0794	1.67E-33	4.53E-17	1.33E-19	3.04E-18
GZMB	0.5275	0.6161	0.5509	0.035	1.51E-30	2.19E-21	1.67E-23	2.89E-22
GPNMB	0.3771	0.4376	0.3863	0.0109	1.77E-14	7.40E-11	2.31E-11	2.93E-11

GDF15	0.4496	0.5422	0.465	0.0191	1.00E-22	2.76E-15	2.27E-16	7.22E-16
FURIN	0.4502	0.5515	0.4704	0.0294	1.33E-23	2.50E-15	9.16E-17	1.13E-15
FSTL3	0.3337	0.4294	0.3545	0.039	6.03E-14	1.11E-08	1.11E-09	5.74E-09
FLT3LG	0.3357	0.3781	0.3503	0.0145	6.55E-11	8.94E-09	1.78E-09	3.68E-09
FAP	0.272	0.3633	0.2909	0.0284	3.94E-10	4.03E-06	7.61E-07	1.54E-06
FABP4	0.1779	0.2387	0.1898	0.0262	5.63E-05	0.00286611	0.00144796	0.00184565
F3	0.4218	0.5871	0.4584	0.0607	3.10E-27	1.84E-13	7.03E-16	2.30E-14
ESM1	0.3883	0.4579	0.4056	0.0277	7.30E-16	1.79E-11	1.78E-12	4.19E-12
ERBB3	0.367	0.4564	0.38	0.0255	9.28E-16	2.55E-10	5.12E-11	1.34E-10
ENPP2	0.2186	0.2633	0.2291	0.0123	8.32E-06	0.00023381	0.00011263	9.88E-05
WFDC2	0.4081	0.5566	0.4447	0.0653	4.26E-24	1.26E-12	5.95E-15	1.69E-13
