# Supplementary material for: TSCytoPred: a deep learning framework for inferring cytokine expression trajectories from irregular longitudinal gene expression data to enhance multi-omics analyses
Source: PeerJ. 2025 Nov 10;13:e20270. doi: 10.7717/peerj.20270 (PMC12614104; doi:10.7717/peerj.20270)
Supplement: Supplemental Information 5 [file peerj-13-20270-s005.pdf]

Supplementary Material S5.

Average prediction performance of TSCytoPred using different interpolation strategies unde[illegible]
5-fold cross validation.

Interploation	R^2	MAE	RMSE	MAPE	CORR
Linear	0.257	0.437	0.610	0.118	0.986
Cubic spline	0.243	0.443	0.618	0.116	0.986
