# Supplementary material for: TSCytoPred: a deep learning framework for inferring cytokine expression trajectories from irregular longitudinal gene expression data to enhance multi-omics analyses
Source: PeerJ. 2025 Nov 10;13:e20270. doi: 10.7717/peerj.20270 (PMC12614104; doi:10.7717/peerj.20270)
Supplement: Supplemental Information 6 — Grid search was adopted for the model tuning, and the hyperparameters showing the best average performance were selected. Row with the bolded font are the hyperparmeters selected. [file peerj-13-20270-s006.pdf]

Supplementary Material S6.
Hyperparameter optimization results for the comparison methods based on the average performance from 5-fold cross-validation. Grid search was adopted for the model tuning, and the hyperparameters showing the best average performance were selected. Row with the bolded font are the hyperparmeters selected.

# of hidden nodes	Learning rate	Batch size	MAE
128-64	1e-4	30	0.5065
256-64	1e-4	30	0.4778
256-128	1e-4	30	0.4364
512-64	1e-4	30	0.4573
512-128	1e-4	30	0.4334
512-256	1e-4	30	0.4263
1024-64	1e-4	30	0.4668
1024-128	1e-4	30	0.4332
1024-256	1e-4	30	0.4253
1024-512	1e-4	30	0.4268
2048-64	1e-4	30	0.4580
2048-128	1e-4	30	0.4337
2048-256	1e-4	30	0.4292
2048-512	1e-4	30	0.4278
2048-1024	1e-4	30	0.4273
256-128-64	1e-4	30	0.4758
512-128-64	1e-4	30	0.4920
512-256-64	1e-4	30	0.4725
512-256-128	1e-4	30	0.4394
1024-128-64	1e-4	30	0.5029
1024-256-128	1e-4	30	0.4347
1024-512-64	1e-4	30	0.4618
1024-512-128	1e-4	30	0.4321
1024-512-256	1e-4	30	0.4259
2048-128-64	1e-4	30	0.4792
2048-256-64	1e-4	30	0.4762
2048-256-128	1e-4	30	0.4268
2048-512-64	1e-4	30	0.4696
2048-512-128	1e-4	30	0.4363
2048-512-256	1e-4	30	0.4246
2048-1024-64	1e-4	30	0.4762
2048-1024-128	1e-4	30	0.4278
2048-1024-256	1e-4	30	0.4262
2048-1024-512	1e-4	30	0.4246
1024-512	1e-2	30	25.9861
1024-512	1e-3	30	0.4823
1024-512	1e-4	30	0.4426

1024-512	1e-5	30	0.4253
1024-512	1e-6	30	0.4847
1024-512	1e-4	10	0.4533
1024-512	1e-4	20	0.4437
1024-512	1e-4	30	0.4253
1024-512	1e-4	40	0.4366
1024-512	1e-4	50	0.4457
1024-512	1e-4	60	0.4367
