# Supplementary material for: TSCytoPred: a deep learning framework for inferring cytokine expression trajectories from irregular longitudinal gene expression data to enhance multi-omics analyses
Source: PeerJ. 2025 Nov 10;13:e20270. doi: 10.7717/peerj.20270 (PMC12614104; doi:10.7717/peerj.20270)
Supplement: Supplemental Information 7 — Grid search was adopted for the model tuning, and the hyperparameters showing the best average performance were selected. Row with the bolded font are the hyperparmeters selected. [file peerj-13-20270-s007.pdf]

Supplementary Material S7.
Hyperparameter optimization results for the comparison methods based on the average performance from 5-fold cross-validation. Grid search was adopted for the model tuning, and the hyperparameters showing the best average performance were selected. Row with the bolded font are the hyperparmeters selected.

Machine learning regression models

Method	Alpha	R^2	MAE	RMSE	MAPE
ElasticNet	10^{-2}	0.081	0.497	0.640	0.134
ElasticNet	**10^{-1}**	**0.247**	**0.449**	**0.585**	**0.125**
ElasticNet	10^{0}	-0.001	0.554	0.698	0.163
ElasticNet	10^{1}	-0.055	0.580	0.726	0.169
Lasso	10^{-2}	0.139	0.483	0.622	0.133
Lasso	**10^{-1}**	**0.207**	**0.465**	**0.603**	**0.131**
Lasso	10^{0}	-0.047	0.575	0.721	0.169
Lasso	10^{1}	-0.055	0.580	0.726	0.169
Ridge	10^{-2}	0.162	0.472	0.610	0.125
Ridge	10^{-1}	0.162	0.472	0.610	0.125
Ridge	10^{0}	0.164	0.471	0.610	0.125
Ridge	**10^{1}**	**0.184**	**0.465**	**0.602**	**0.123**

Deep learning models

	# of channels	Layer 1	Layer 2	MAE
LSTM	-	250	125	0.588
LSTM	-	500	125	0.588
LSTM	-	500	250	0.587
LSTM	-	1000	125	0.585
LSTM	-	1000	250	0.585
LSTM	-	1000	500	0.585
LSTM	-	2000	125	0.583
LSTM	-	2000	250	0.586
LSTM	-	2000	500	0.526
LSTM	-	2000	1000	**0.514**
CNN-LSTM	250	125	60	0.542
CNN-LSTM	500	250	125	0.482
CNN-LSTM	1000	500	125	0.489
CNN-LSTM	1000	500	250	0.581
CNN-LSTM	2000	1000	125	0.696
CNN-LSTM	2000	1000	250	**0.514**
CNN-LSTM	2000	1000	500	0.586
CNN-LSTM	4000	2000	125	0.587
CNN-LSTM	4000	2000	250	0.585
CNN-LSTM	4000	2000	500	0.582
CNN-LSTM	4000	2000	1000	0.583
