# Supplementary material for: TSCytoPred: a deep learning framework for inferring cytokine expression trajectories from irregular longitudinal gene expression data to enhance multi-omics analyses
Source: PeerJ. 2025 Nov 10;13:e20270. doi: 10.7717/peerj.20270 (PMC12614104; doi:10.7717/peerj.20270)
Supplement: Supplemental Information 8 [file peerj-13-20270-s008.pdf]

Supplementary Material S8.

Average prediction performance results with 95% confidence interval of TSCytoPred with other comparison methods based on the 5-fold cross validation.

Metric	TSCytoPred	NN	ElasticNet	Lasso	Ridge	Linear	LSTM	CNN-LSTM	ARIMA
R^2	0.257 ± 0.057	0.246 ± 0.063	0.247 ± 0.040	0.207 ± 0.044	0.184 ± 0.065	0.161 ± 0.069	-0.099 ± 0.079	0.175 ± 0.089	-1.783 ± 0.883
MAE	0.437 ± 0.026	0.442 ± 0.025	0.449 ± 0.024	0.465 ± 0.024	0.465 ± 0.023	0.472 ± 0.023	0.583 ± 0.031	0.465 ± 0.038	0.829 ± 0.094
RMSE	0.610 ± 0.038	0.618 ± 0.040	0.620 ± 0.034	0.639 ± 0.037	0.641 ± 0.033	0.650 ± 0.033	0.801 ± 0.054	0.651 ± 0.062	1.052 ± 0.137
MAPE	0.118 ± 0.014	0.116 ± 0.011	0.125 ± 0.015	0.131 ± 0.016	0.123 ± 0.018	0.125 ± 0.019	0.171 ± 0.027	0.121 ± 0.021	6.6E+04 ± 1.1E+05
CORR	0.986 ± 0.002	0.985 ± 0.002	0.985 ± 0.002	0.984 ± 0.002	0.984 ± 0.002	0.984 ± 0.002	0.983 ± 0.003	0.989 ± 0.002	0.366 ± 0.090
