# Supplementary material for: TSCytoPred: a deep learning framework for inferring cytokine expression trajectories from irregular longitudinal gene expression data to enhance multi-omics analyses
Source: PeerJ. 2025 Nov 10;13:e20270. doi: 10.7717/peerj.20270 (PMC12614104; doi:10.7717/peerj.20270)
Supplement: Supplemental Information 9 [file peerj-13-20270-s009.pdf]

Supplementary Material S9.

Average prediction performance results of TSCytoPred and comparison methods based on 3-fold cross-validation.

Metric	TSCytoPred	NN	Linear	Ridge	ElasticNet	Lasso	CNN-LSTM
R^2	0.274	0.239	0.191	0.209	0.253	0.218	-0.031
MAE	0.438	0.451	0.471	0.465	0.455	0.469	0.570
RMSE	0.610	0.629	0.613	0.606	0.594	0.610	0.784
MAPE	0.115	0.116	0.122	0.121	0.127	0.133	0.162
CORR	0.990	0.989	0.989	0.989	0.989	0.989	0.984
