# Supplementary material for: TSCytoPred: a deep learning framework for inferring cytokine expression trajectories from irregular longitudinal gene expression data to enhance multi-omics analyses
Source: PeerJ. 2025 Nov 10;13:e20270. doi: 10.7717/peerj.20270 (PMC12614104; doi:10.7717/peerj.20270)
Supplement: Supplemental Information 10 [file peerj-13-20270-s010.pdf]

Supplementary Material S10.

Average prediction performance results of TSCytoPred and comparison methods based on 5-fold cross-validation after removing outlier cytokines.

Average performance after removing outlier cytokines with outliers in >5% of samples

Metric	TSCytoPred	NN	Linear	Ridge	ElasticNet	Lasso	CNN-LSTM
R^2	0.272	0.263	0.183	0.206	0.264	0.221	0.193
MAE	0.428	0.434	0.461	0.454	0.440	0.458	0.457
RMSE	0.589	0.598	0.594	0.586	0.570	0.590	0.632
MAPE	0.110	0.111	0.119	0.118	0.120	0.127	0.113
CORR	0.991	0.991	0.990	0.990	0.991	0.990	0.989

Average performance after removing outlier cytokines with outliers in >3% of samples

Metric	TSCytoPred	NN	Linear	Ridge	ElasticNet	Lasso	CNN-LSTM
R^2	0.318	0.307	0.203	0.230	0.276	0.230	0.170
MAE	0.428	0.414	0.453	0.455	0.436	0.455	0.464
RMSE	0.576	0.560	0.583	0.582	0.560	0.582	0.640
MAPE	0.102	0.106	0.120	0.129	0.122	0.129	0.117
CORR	0.992	0.992	0.990	0.990	0.991	0.990	0.989
