# Supplementary material for: TSCytoPred: a deep learning framework for inferring cytokine expression trajectories from irregular longitudinal gene expression data to enhance multi-omics analyses
Source: PeerJ. 2025 Nov 10;13:e20270. doi: 10.7717/peerj.20270 (PMC12614104; doi:10.7717/peerj.20270)
Supplement: Supplemental Information 11 [file peerj-13-20270-s011.pdf]

Supplementary Material S11.

Average prediction performance results of TSCytoPred with other comparison methods based on the 5-fold cross validation using COVID-19 patients with two time points.

Metric	TSCytoPred	NN	Linear	Ridge	ElasticNet	Lasso	CNN-LSTM
R^2	0.240	0.232	2.54E+23	0.160	0.236	0.196	0.049
MAE	0.436	0.439	1.15E+11	0.465	0.449	0.464	0.518
RMSE	0.610	0.615	1.55E+11	0.604	0.584	0.601	0.717
MAPE	0.115	0.116	2.66E+10	0.122	0.124	0.132	0.144
CORR	0.990	0.990	0.209	0.989	0.990	0.989	0.986
