# Supplementary material for: TSCytoPred: a deep learning framework for inferring cytokine expression trajectories from irregular longitudinal gene expression data to enhance multi-omics analyses
Source: PeerJ. 2025 Nov 10;13:e20270. doi: 10.7717/peerj.20270 (PMC12614104; doi:10.7717/peerj.20270)
Supplement: Supplemental Information 12 [file peerj-13-20270-s012.pdf]

Supplementary Material S12.

Average inference performance results using original three–time point cohort by predicting cytokine abundance dynamics in reverse based on the 5-fold cross validation.

Metric	TSCytoPred	NN	Linear	Ridge	ElasticNet	Lasso	CNN-LSTM
R^2	0.250	0.242	0.161	0.184	0.247	0.207	0.083
MAE	0.441	0.442	0.472	0.465	0.449	0.465	0.502
RMSE	0.616	0.615	0.611	0.602	0.585	0.603	0.698
MAPE	0.119	0.116	0.125	0.123	0.125	0.131	0.138
CORR	0.990	0.990	0.989	0.989	0.990	0.989	0.987
