# Supplementary material for: TSCytoPred: a deep learning framework for inferring cytokine expression trajectories from irregular longitudinal gene expression data to enhance multi-omics analyses
Source: PeerJ. 2025 Nov 10;13:e20270. doi: 10.7717/peerj.20270 (PMC12614104; doi:10.7717/peerj.20270)

Supplementary Material S13. Distribution of actual and inferred cytokine expression in test data for COVID-19 dataset using different time-series regression methods. (Notations: ns- not significant, * - p-value < 0.05, ** - p-value < 0.01.)

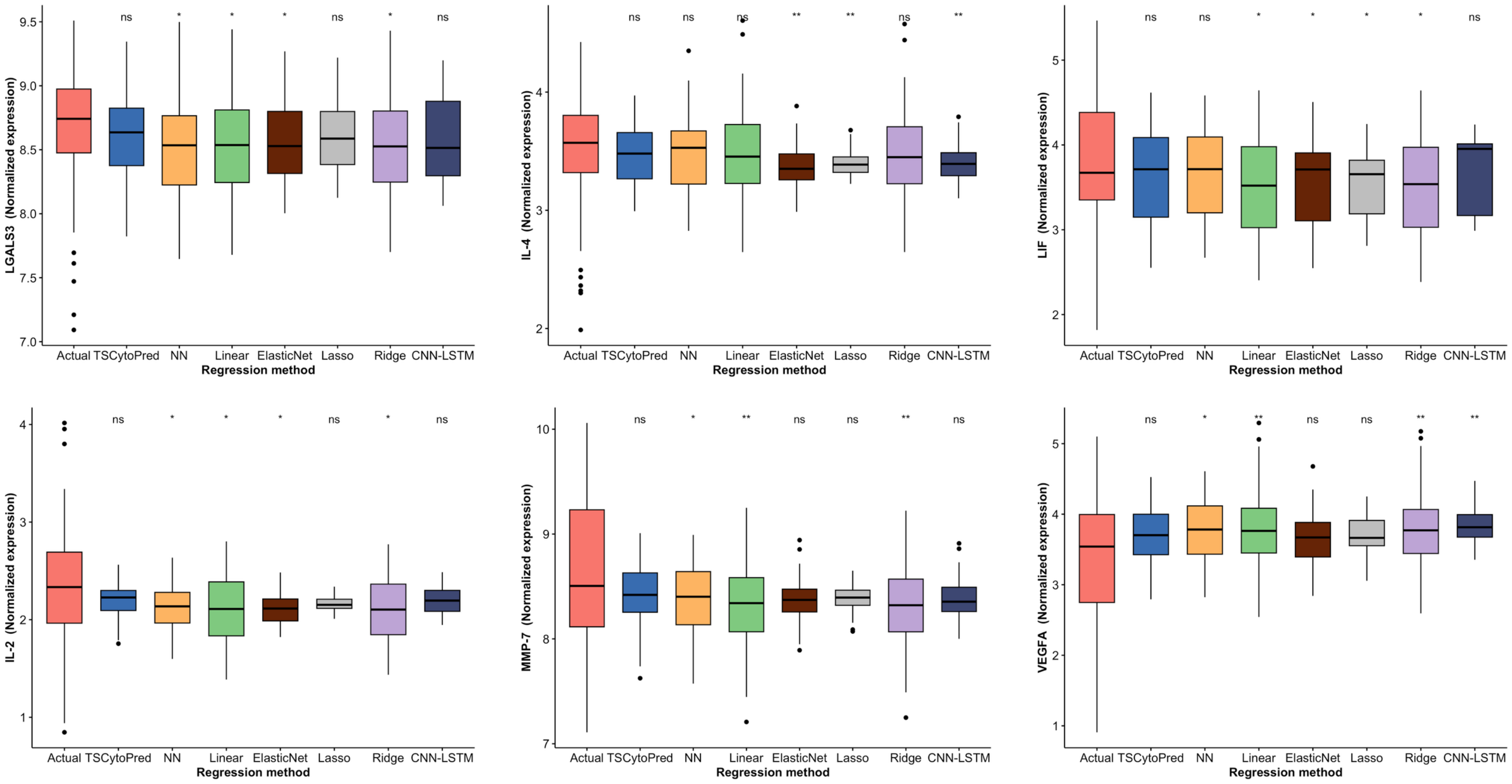

Supplement: Supplemental Information 13 — (Notations: ns- not significant, * - p-value ¡ 0.05, ** - p-value ¡ 0.01.) [file peerj-13-20270-s013.pdf]
