# Supplementary material for: TSCytoPred: a deep learning framework for inferring cytokine expression trajectories from irregular longitudinal gene expression data to enhance multi-omics analyses
Source: PeerJ. 2025 Nov 10;13:e20270. doi: 10.7717/peerj.20270 (PMC12614104; doi:10.7717/peerj.20270)
Supplement: Supplemental Information 14 [file peerj-13-20270-s014.pdf]

Supplementary Material S14.

Average prediction performance results with 95% confidence interval of TSCytoPred under different feature sets based on the 5-fold cross validation.

Metric	CytReg	CytReg + STRING
# of genes	242	875
R^2	0.097 ± 0.098	0.134 ± 0.064
MAE	0.487 ± 0.043	0.476 ± 0.028
RMSE	0.676 ± 0.081	0.659 ± 0.042
MAPE	0.128 ± 0.028	0.125 ± 0.014
CORR	0.988 ± 0.001	0.989 ± 0.001

	CytReg + STRING + CORR			
Metric	Top 10	Top 20	Top 30	Top 50
# of genes	1569	2141	2628	3429
R^2	0.238 ± 0.064	0.244 ± 0.061	0.245 ± 0.067	0.257 ± 0.057
MAE	0.444 ± 0.025	0.442 ± 0.025	0.443 ± 0.028	0.437 ± 0.026
RMSE	0.621 ± 0.038	0.618 ± 0.039	0.619 ± 0.041	0.610 ± 0.038
MAPE	0.115 ± 0.011	0.116 ± 0.011	0.116 ± 0.008	0.118 ± 0.014
CORR	0.990 ± 0.001	0.990 ± 0.001	0.990 ± 0.001	0.986 ± 0.002

	CytReg + STRING + MI			
Metric	Top 10	Top 20	Top 30	Top 50
# of genes	2086	3131	4021	5615
R^2	0.162 ± 0.086	0.193 ± 0.072	0.195 ± 0.070	0.201 ± 0.073
MAE	0.463 ± 0.025	0.456 ± 0.028	0.456 ± 0.023	0.454 ± 0.022
RMSE	0.642 ± 0.041	0.634 ± 0.042	0.633 ± 0.038	0.632 ± 0.037
MAPE	0.120 ± 0.015	0.12 ± 0.013	0.119 ± 0.012	0.120 ± 0.009
CORR	0.990 ± 0.001	0.999 ± 0.001	0.990 ± 0.001	0.990 ± 0.001
