# Supplementary material for: TSCytoPred: a deep learning framework for inferring cytokine expression trajectories from irregular longitudinal gene expression data to enhance multi-omics analyses
Source: PeerJ. 2025 Nov 10;13:e20270. doi: 10.7717/peerj.20270 (PMC12614104; doi:10.7717/peerj.20270)
Supplement: Supplemental Information 15 [file peerj-13-20270-s015.pdf]

Supplementary Material S15.

Average prediction performance results of TSCytoPred with other comparison methods based on the 5-fold cross validation using COVID-19 patients with two time points.

Testing scenario	w/o infer	w/ infer (TSCytoPred)	w/ infer (NN)	w/ infer (Linear)	w/ infer (ElasticNet)	w/ infer (Lasso)	w/ infer (Ridge)	w/ infer (CNN-LSTM)
Timepoint-level	0.565 ± 0.028	0.602 ± 0.042	0.570 ± 0.051	0.596 ± 0.053	0.540 ± 0.037	0.547 ± 0.041	0.562 ± 0.072	0.573 ± 0.061
Patient-worst-level	0.568 ± 0.034	0.592 ± 0.115	0.565 ± 0.121	0.508 ± 0.101	0.526 ± 0.164	0.550 ± 0.126	0.528 ± 0.108	0.527 ± 0.190
